# Capacity of tTreg generation is not impaired in the atrophied thymus

**DOI:** 10.1371/journal.pbio.2003352

**Published:** 2017-11-08

**Authors:** Jiyoung Oh, Weikan Wang, Rachel Thomas, Dong-Ming Su

**Affiliations:** Department of Microbiology, Immunology, & Genetics, University of North Texas Health Science Center, Fort Worth, Texas, United States of America; National Cancer Institute, United States of America

## Abstract

Postnatal thymic epithelial cell (TEC) homeostatic defect- or natural aging-induced thymic atrophy results in a decline in central T-cell tolerance establishment, which is constituted by thymocyte negative selection and cluster of differentiation (CD) 4^+^ thymic regulatory T (tTreg) cell generation. Emerging evidence shows this decline mainly results from defects in negative selection, but there is insufficient evidence regarding whether tTreg cell generation is also impaired. We mechanistically studied tTreg cell generation in the atrophied thymus by utilizing both postnatal TEC-defective (resulting from *FoxN1-*floxed conditional knockout [cKO]) and naturally aged mouse models. We found that the capacity of tTreg cell generation was not impaired compared to CD4^+^ thymic conventional T cells, suggesting thymic atrophy positively influences tTreg cell generation. This is potentially attributed to decreased T cell receptor (TCR) signaling strength due to inefficiency in promiscuous expression of self-antigens or presenting a neo-self-antigen by medullary TECs, displaying decreased negative selection-related marker genes (Nur77 and CD5^high^) in CD4 single positive (SP) thymocytes. Our results provide evidence that the atrophied thymus attempts to balance the defective negative selection by enhancing tTreg cell generation to maintain central T-cell tolerance in the elderly. Once the balance is broken, age-related diseases could take place.

## Author summary

Chronic inflammation in the elderly is partially attributed to atrophy of the thymus—an organ that regulates the immune system—and in particular the ability of organisms to recognize their own cells—a phenomenon known as central tolerance. Immune central tolerance is established by two processes: first, immune cells that react strongly to self are eliminated in a process called negative selection, and second, thymic regulatory T (tTreg) cells are generated to suppress self-reactive immune reactions. The former has already been reported to be defective in the aged thymus, but whether the generation of new tTreg cells is also impaired has remained unclear. Here, we analyzed the effect of aging on tTreg cell generation and found that the atrophied thymus is still able to make new tTreg cells; indeed, we show that tTreg cell generation capacity is enhanced when compared with other naïve T cells from the same thymus. We conclude that the balance of defective negative selection with enhanced tTreg cell generation may be necessary to avoid autoimmune diseases during aging.

## Introduction

Central T-cell tolerance, which refers to the mechanism by which newly developing T cells are rendered nonreactive to self [1], plays an important role in controlling autoimmunity [2]. Central T-cell tolerance is established in the thymus through two main processes: thymocyte negative selection to deplete self-reactive T clones [3, 4] and tTreg cell generation to execute suppressive function in the periphery [5, 6]. Both negative selection and tTreg cell generation are critically dependent on medullary thymic epithelial cell (mTEC)-presentation (promiscuous expression) of self-antigens/peptides binding to the MHC groove (self-peptide-Major Histocompatibility Complex [self-pMHC]) [4, 7]. Once the self-pMHC interacts with T cell receptors (TCRs) on the self-reactive T clones, a TCR signal is produced. Based on affinity/avidity of self-pMHC to self-TCR, this interaction may produce a strong signal, causing negative selection to lead to deletion of this self-T clone. However, if a weak signal is produced, it will allow this T clone to survive and differentiate into thymic conventional T (tTcon) cells [8]. Moreover, if the TCR receives a signal at an intermediate strength, or a strong but transient signal (hit-and-run), this self-T clone will undergo tTreg cell differentiation [9, 10]. Therefore, central T-cell tolerance establishment in the thymus is determined by the interaction between the self-pMHC on the thymic epithelial cells (TECs) and TCR on the developing thymocytes.

During aging, the thymus undergoes a progressively age-related atrophy, or involution, which leads to the impairment of central T-cell tolerance establishment, resulting in increased self-reactivity in the periphery, which is one of the etiologies that induce the manifestation of chronic inflammation in the elderly (termed inflammaging) [11]. This atrophy is attributed both to the deterioration of TEC homeostasis, particularly mTEC, regulated by the *FOXN1* (Forkhead box protein N1) gene [12], and in part, the decline of the autoimmune regulator *Aire* gene in mTECs, which causes a prominent dysfunction in negative selection [13, 14]. The generation of tTreg cells are critical for the maintenance of self-antigen tolerance and immune homeostasis [6]. Although evidence shows that aging is associated with enhancement of the peripheral regulatory T cell (pTreg) population as well as its function [15, 16], it is largely unclear whether the process of tTreg cell development in the age-related atrophied thymus is enhanced or impaired. Although newly generated tTreg cells were reported to be substantially declined with age in a recent report [17], the experimental evidence on this point is still insufficient. Therefore, the question is whether tTreg cell generation in the aged thymus exhibits any decline or enhancement along with declined negative selection; whether thymic atrophy differentially impacts these two processes of central T-cell tolerance establishment; and what the underlying mechanism is.

In this study, we explored how tTreg cell generation in the atrophied thymus is affected and by what mechanism in mouse models. We demonstrated that although thymic atrophy perturbs negative selection, resulting in an inability to sufficiently deplete self-reactive T clones, it was compensated for by relative enhancement of tTreg cell generation, as shown by an increased ratio of percentage of tTreg cells to percentage of tTcon cells and unreduced absolute tTreg cell numbers despite a dramatically decreased whole thymic cellularity. This relative enhancement was attributed to the new generation/differentiation of tTreg cells, rather than any changes in apoptotic or anti-apoptotic features, which was demonstrated by enhanced phosphoralated-Zap70 (p-Zap70) and unchanged apoptotic (Annexin V^+^) cells in certain cell subsets. We determined that the mechanism is potentially due to a declined TCR signaling strength, shifting from high strength, which is required for negative selection, to intermediate strength, which favors tTreg cell generation, because negative selection-related marker gene signals (Nur77 and cluster of differentiation [CD] 5^high^) were decreased in certain cell subsets of the atrophied thymus compared to those in the young thymus. Additionally, this declined TCR signaling strength is due to an inefficient presentation of self-pMHC on mTECs, which was demonstrated in a neo/mock self-antigen rat insulin promoter (RIP)-driven membrane-bound ovalbumin (mOVA) transgenic (Tg) mouse model. Together, our results provide evidence that the atrophied thymus attempts to balance the defective negative selection by relatively enhancing tTreg cell generation to maintain central T-cell tolerance in the elderly.

## Results

### Thymic atrophy relatively enhanced tTreg generation associated with a reduced mature Treg thymic reentrance

**Fig 1:**
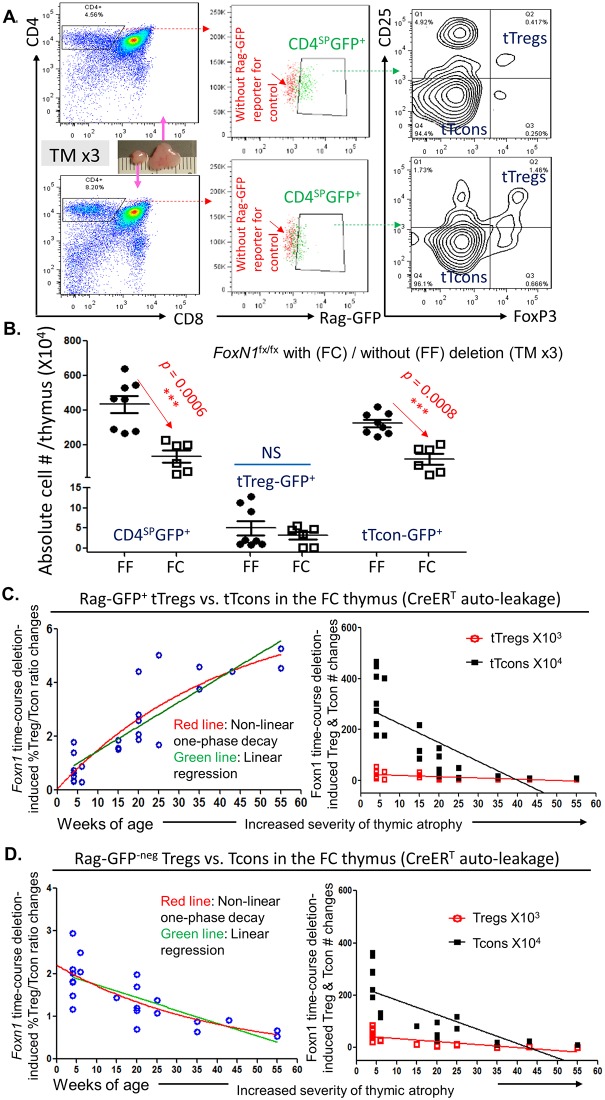
Relatively enhanced activity of new (Rag-GFP^+^) tTreg cell generation along with reduced thymic-reentered (Rag-GFP^-neg^) Treg cells were related to the severity of thymic atrophy. **(A)** A representative flow cytometric gate strategy from young mice of FF (normal thymus, top panels) or FC (atrophied thymus, bottom panels) with a Rag-GFP reporter shows gates of CD4^SP^ (left panels), CD4^SP^-GFP^+^ (middle panels), and tTcon cells and tTreg cells in the CD4^SP^-GFP^+^ population from the same thymus (right panels). **(B)** Summarized absolute cell numbers per thymus from panel-A of newly generated (Rag-GFP^+^) CD4^SP^, tTreg cells, and tTcon cells from FF-Rag-GFP and FC-Rag-GFP mice treated with TM x3 (for induction of acute *FoxN1*^fx/fx^ gene deletion), respectively. A Student *t* test was used to determine statistical significance between groups. **(C)** *FoxN1*^fx/fx^ time-course deletion (due to the CreER^T^ autoleakage over time) induced dynamic changes in ratio (left panel) and absolute cell numbers per thymus (right panel) of newly generated (Rag-GFP^+^) tTreg cells and tTcon cells in the same thymus. **(D)** Potential peripheral thymic-reentered (Rag-GFP^-neg^) Treg cells and Tcon cells in the same thymus. Two curves in left panels of C and D show linear regression (green lines) and nonlinear one-phase decay (red lines), respectively. Lines in right panels of C and D are linear regression, while data presented by nonlinear one-phase decay are shown in S2A Fig. GraphPad-5 statistic software was used for analysis. Each symbol represents an individual animal sample. Underlying data used in the generation of this figure can be found in S1 Data. CreER^T^, ubiquitous promoter-driven Cre-recombinase and estrogen-receptor fusion protein; FC, *FoxN1*^fx/fx^/CreER^T^; FF, *FoxN1*^fx/fx^ without CreER^T^ for controls; *Foxn1*, Forkhead box protein N1; *FoxN1*^fx/fx^, *loxp*-flanked *FoxN1* gene; GFP, green fluorescent protein; Tcon, conventional T cell; TM, tamoxifen; tTcon, thymic conventional T cell; tTreg, thymic regulatory T cell.

**Fig 2:**
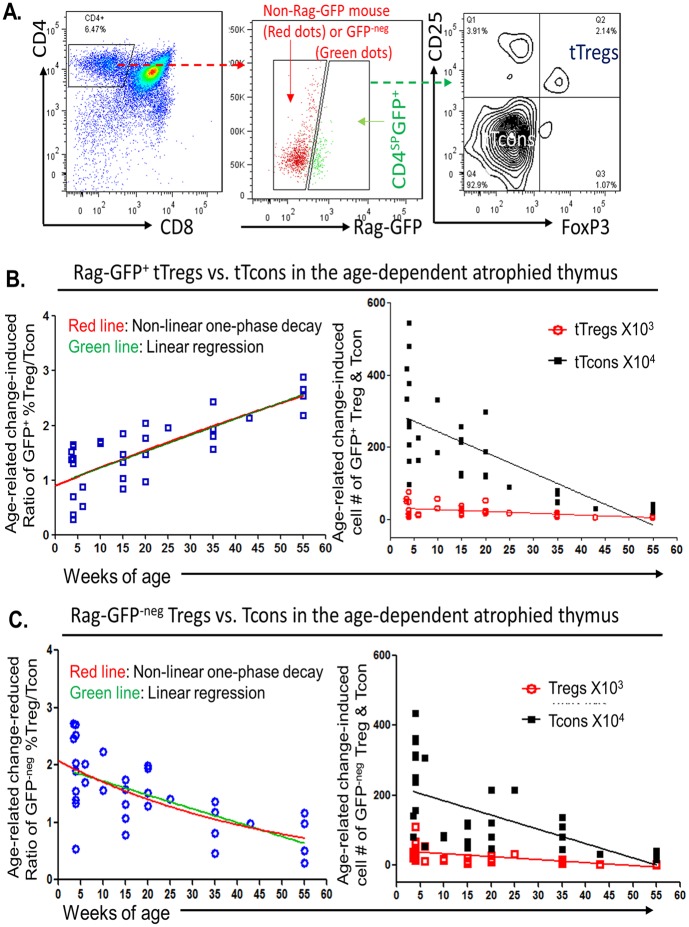
Activity of new (Rag-GFP^+^) tTreg cell generation was relatively enhanced, while thymic-reentering (Rag-GFP^-neg^) Treg cells were declined in an age-dependent manner in the naturally aged thymus. **(A)** A representative flow cytometric gate strategy from Rag-GFP reporter mice shows gates of CD4^SP^ (left panel), CD4^SP^-Rag-GFP^+^ and CD4^SP^-Rag-GFP^-neg^ (middle panel, the red dots are cells from non-Rag-GFP reporter mouse for setting up a cutoff), and tTcon cells and tTreg cells in the CD4^SP^-Rag-GFP^+^ population (right panel). **(B)** In newly generated (Rag-GFP^+^) CD4^SP^ population, age-dependent dynamic changes in ratio of tTreg cells versus tTcon cells (left panel) and absolute cell numbers of tTreg cells and tTcon cells per thymus (right panel). **(C)** Age-dependent dynamic changes in ratio (left panel) and absolute cell numbers per thymus (right panel) of thymic-reentering (Rag-GFP^-neg^) Treg cells and Tcon cells. Two curves in left panels of B and C show linear regression (green lines) and nonlinear one-phase decay (red lines), respectively. Lines in right panels of B and C are linear regression, while data presented by nonlinear one-phase decay are shown in S2B Fig. GraphPad-5 statistic software was used for analysis. Each symbol represents an individual animal sample. Underlying data used in the generation of this figure can be found in S1 Data. CD, cluster of differentiation; GFP, green fluorescent protein; Tcon, conventional T cell; TM, tamoxifen; Treg, regulatory T cell; tTcon, thymic conventional T cell; tTreg, thymic regulatory T cell.

To determine whether the capacity of newly generated tTreg cells is impaired in aged thymus [17] and/or in the TEC defect-induced atrophied thymus, we utilized our previously generated mouse model with accelerated thymic atrophy (due to postnatal TEC homeostatic defect) [12], in which the *loxp*-flanked *FoxN1* gene (*FoxN1*^fx/fx^) [18] can be deleted by ubiquitous promoter-driven Cre-recombinase and estrogen-receptor fusion protein (CreER^T^) mediation through either a tamoxifen (TM) induction or a CreER^T^ autoleakage with age. We named this model the FC (*FoxN1*^fx/fx^/CreER^T^) mouse and FF (*FoxN1*^fx/fx^ without CreER^T^ for controls) mouse. One-month-old FC thymi without any TM treatment exhibit the same thymocyte profiles as those in wild-type (WT) young mice. However, when young FC mice are treated with TM for 3 consecutive days (TM x3), or FC mice are housed for more than 6 months without any TM treatment but with an age-related CreER^T^ autoleakage, their thymi exhibit thymic atrophy analogous to aged (approximately 18-month-old) WT thymi (details in S1 Fig). In addition, a *rag*-*gfp* reporter gene was utilized in this model to mark newly generated thymocytes [14], which we termed either FC-Rag-GFP or FF-Rag-GFP mice. We first observed absolute cell numbers of newly generated (Rag-GFP^+^) CD4 single positive (SP) (CD4^SP^) thymocytes, including tTreg cells and tTcon cells in CD4^SP^ population, in the acute atrophied thymus (TM x3) (Fig 1A and 1B). We found that although thymic mass was dramatically reduced (Fig 1A image) with reduced numbers of newly generated CD4^SP^ and tTcon cells, the newly generated tTreg cells were not decreased (Fig 1B), implying tTreg cells are unaffected by the acute thymic atrophy. We then analyzed integration of tTreg cells and tTcon cells in newly generated CD4^SP^ population by measuring the age-dependent dynamic ratios of percentage of tTreg cells to percentage of tTcon cells in the accelerated atrophied thymus (*FoxN1*^fx/fx^ deletion through a CreER^T^ autoleakage over time [12]). The ratio was increased rather than decreased (Fig 1C, left panel). Under the background of a dramatically decreased total thymic cell number in the atrophied thymus, the absolute cell numbers of newly generated tTcon cells were dynamically decreased, while tTreg cell numbers were not (Fig 1C, right panel). Additionally, we gated the Rag-GFP reporter negative (Rag-GFP^-neg^) CD4^SP^ population (gate shown in Fig 2A), which are not composed of newly generated T cells and were proposed to be primarily peripheral cells that had recirculated back to the thymus (i.e., thymic-reentered mature T cells) [17]. These thymic-reentered pTreg cells were also proposed to suppress development of newly generated tTreg cells [17]. The ratio of percentage of regulatory T cells (Treg) (mainly pTreg cells [17]) to percentage of conventional T (Tcon) cells of Rag-GFP^-neg^ in the thymus was decreased with age (Fig 1D, left panel), while the absolute cell numbers of both Rag-GFP^-neg^ populations (Fig 1D, right panel) mirrored the pattern observed with the Rag-GFP^+^ population (Fig 1C, right panel). Together, these results suggest that the severity of thymic atrophy positively influences new tTreg cell generation and negatively affects pTreg cells reentering the thymus.

In the naturally aged (WT, ≥ 18 months old) mouse, pTreg cells are increased [15] due to their accumulation, which is attributed to decreased *Bim* (a pro-apoptotic gene) expression [19, 20]. However, the *FoxN1*^fx/fx^-deleted mice have a relative young periphery since only the thymus undergoes an accelerated aging. Therefore, the young FC mouse does not have increased/accumulated pTreg cells. In order to account for the peripheral interference of new tTreg cell generation in the naturally age-related atrophied thymus, we investigated the same parameters in WT (containing Rag-GFP reporter) mouse thymi (Fig 2). As we expected, in the population of new Rag-GFP^+^CD4^SP^ cells (Fig 2A, left and middle panels), the ratio of percentage of tTreg cells to percentage of tTcon cells was shown also to be increased in an age-dependent manner (Fig 2B, left panel). The absolute cell numbers of either tTreg cells or tTcon cells (Fig 2B, linear regression in right panel; for nonlinear one-phase decay, see S2 Fig) showed the same tendency as FC mice in Fig 1C right panel, as did the Rag-GFP^-neg^CD4^SP^ populations, i.e., potential thymic-reentered cells (Fig 2C). These results are consistent with the findings in FC mice shown in Fig 1. Therefore, we concluded that thymic atrophy does not impair the capacity of new tTreg cell generation and is associated with a reduction of pTreg cells reentering in an age-dependent manner.

### Relatively enhanced tTreg cell generation in the atrophied thymus was driven by the thymic microenvironment, rather than tTreg cell intrinsic alterations

**Fig 3:**
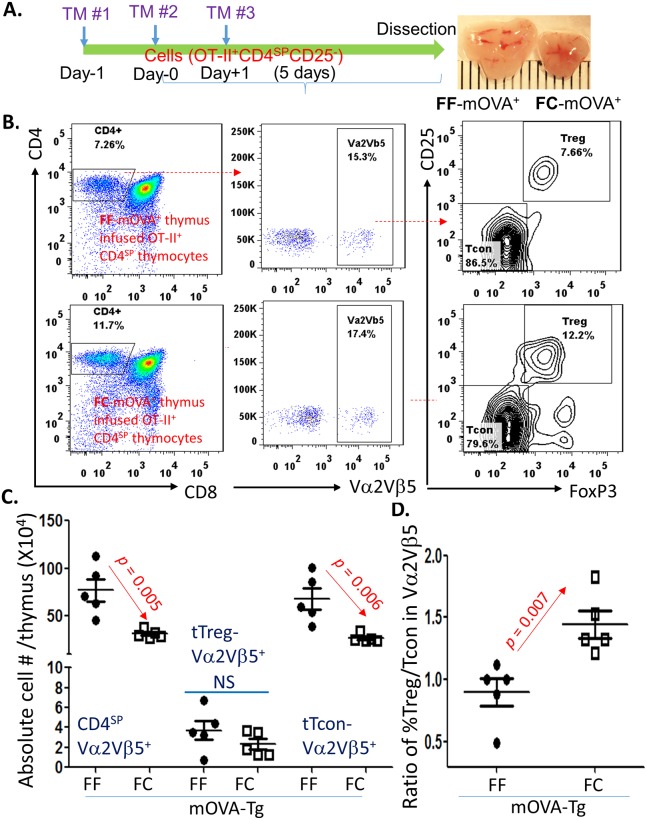
Acute atrophied thymic microenvironment enhanced intrathymic generation of OT-II^+^ TCR-Tg tTreg cells in the FC-mOVA-Tg mouse thymus. **(A)** A schematic workflow shows that young FF-mOVA-Tg (normal thymus) or FC-mOVA-Tg (atrophied thymus) mice were i.t. infused with sorted CD4^SP^CD25^-neg^ thymocytes (pre-tTreg cells) from OT-II^+^/Rag ^−/−^ mice. These mice were injected with TM x3 i.p. at Day−1, Day0, and Day+1 to induce *FoxN1*^fx/fx^ deletion under a CreER^T^ mediation. Five days after the i.t. infusion of CD4^SP^CD25^-neg^ pre-tTreg cells, the thymi of recipients were isolated and analyzed for infused OT-II^+^ TCR-Tg thymocyte-derived tTreg cells and tTcon cells with flow cytometry. **(B)** A representative flow cytometric gate strategy shows gates of CD4^SP^ (left panels), CD4^SP^-Vα2^+^Vβ5^+^ (mainly from infused OT-II TCR-Tg thymocytes) (middle panels), and tTcon cells versus tTreg cells (right panels) in FF-mOVA-Tg (top panels) and FC-mOVA-Tg (bottom panels) mice. **(C)** Summarized absolute cell numbers of CD4^SP^-Vα2^+^Vβ5^+^, tTreg cells, and tTcon cells in the FF-mOVA-Tg and FC-mOVA-Tg mice. **(D)** The ratios of Vα2^+^Vβ5^+^ percentage of tTreg cells versus percentage of tTcon cells in FF-mOVA-Tg and FC-mOVA-Tg thymi, respectively. A Student *t* test was used to determine statistical significance between groups, and all level bars in panels C and D are expressed as mean ± SEM. Each symbol represents an individual animal sample. Underlying data used in the generation of this figure can be found in S1 Data. CD, cluster of differentiation; CreER^T^, ubiquitous promoter-driven Cre-recombinase and estrogen-receptor fusion protein; FC, *FoxN1*^fx/fx^/CreER^T^; FF, *FoxN1*^fx/fx^ without CreER^T^ for controls; *Foxn1*, Forkhead box protein N1; *FoxN1*^fx/fx^ or *FoxN1-*floxed, *loxp*-flanked *FoxN1* gene; i.p., intraperitoneally; i.t., intrathymically; mOVA, membrane-bound ovalbumin; NS, not significant; TCR, T cell receptor; Tg, transgenic; TM, tamoxifen; tTcon, thymic conventional T cell; tTreg, thymic regulatory T cell.

We believe that the atrophied thymic microenvironment promotes the activity of new tTreg cell generation. To exclude any intrinsic alterations from tTreg cells in the atrophied thymus, such as tTreg cells acquiring any refractory behavior for anti-apoptosis or loss of suppressive function, we designed an adaptive transfer experiment based on a published protocol [21]. We intrathymically (i.t.) injected sorted pre-tTreg cells (CD4^SP^CD25^-neg^ thymocytes) from a pool of young OT-II^+^ TCR Tg (MHC class-II restricted ovalbumin-specific TCR transgenic) mice [22] (with *Rag*^−/−^ background, details in S3 Fig) into either normal (FF control) or acute atrophied (FC, *FoxN1*^fx/fx^CreER^T^ with a TM x3 induction) thymi containing RIP-mOVA Tg TECs [23, 24], termed FC- or FF-mOVA mice (Fig 3A). In this setting, with an interaction between mOVA-MHC and OT-II^+^ TCR, these grafted OT-II^+^ (bearing Vα2 and Vβ5 TCR subunits which recognize mOVA antigen) CD4^SP^ clones from young mice, which do not have any aging-related imprints or characteristics, undergo either negative selection-driven apoptosis or tTreg cell development-related differentiation, dependent on TCR signaling strength produced by the interaction [25, 26].

We first verified that the atrophied thymic microenvironment promoted the grafted pre-tTreg cells (CD4^SP^CD25^-neg^ thymocytes) to differentiate into tTreg cells in an increased proportion compared to tTcon cells (Fig 3B bottom panels and Fig 3D) and that there were unchanged absolute cell numbers (Fig 3C) compared to the counterpart in the FF-mOVA normal thymic microenvironment. The results therefore confirm that changes in the thymic microenvironment, rather than changes intrinsic in T clones, enhance the differentiation toward tTreg cells.

**Fig 4:**
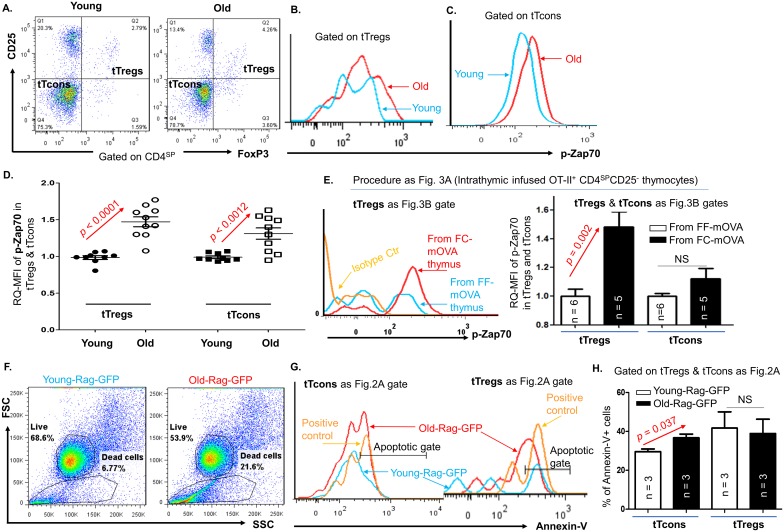
p-Zap70 was increased in both tTreg cells and tTcon cells, but apoptosis was only increased in tTcon cells in the aged and atrophied thymus. **(A)** Representative gate strategy shows the percentage of tTreg cells versus tTcon cells from the thymi of WT young and naturally aged mice. **(B)** A representative histogram shows expression of p-Zap70 in the tTreg cells of WT young (blue curve) and naturally aged (red curve) mice. **(C)** A representative histogram shows expression of p-Zap70 in the tTcon cells of WT young (blue curve) and naturally aged (red curve) mice. **(D)** A summary of RQ-MFI of p-Zap70 in the tTreg cells and tTcon cells of WT young and naturally aged mice. **(E)** The workflow is the same as Fig 3A. Left panel: A representative result shows expression of p-Zap70 in the tTreg cells derived from i.t. infused sorted CD4^SP^CD25^-neg^ pre-tTreg cell thymocytes of OT-II^+^/Rag^−/−^ mice in FF-mOVA (blue line) and FC-mOVA (red line) thymi. Right panel: A summary of RQ-MFI of p-Zap70 in the tTreg cells and tTcon cells from left penal mice. **(F)** Representative dot plots from young and old-Rag-GFP reporter mice show increased dead/necrotic cells in the old mice. **(G)** Histogram shows Annexin-V^+^ apoptotic cells in tTcon cells and tTreg cells (gates as in Fig 2A right panel). **(H)** A summarized result of percentages of Annexin-V^+^ apoptotic tTcon cells and tTreg cells in young and old-Rag-GFP mice, respectively. In panels D, E, and H, Student *t* test was used to determine statistical significance between groups; data are expressed as mean ± SEM; and each symbol represents an individual animal sample or *n* = animal numbers. Underlying data used in the generation of this figure (panels D, E, and H) can be found in S1 Data. CD, cluster of differentiation; FF, *FoxN1*^fx/fx^ without CreER^T^ for controls; i.t., intrathymically; mOVA-Tg, membrane-bound ovalbumin transgenic mouse model; OT-II^+^ TCR Tg, MHC class-II restricted ovalbumin-specific TCR transgenic; mOVA, membrane-bound ovalbumin; p-Zap70, phosphorylated-zeta-chain-associated protein kinase 70; RQ-MFI, relative quantitative Mean Fluorescence Intensity; tTcon, thymic conventional T cell; tTreg, thymic regulatory T cell; WT, wild-type.

We then observed an increased intensity of p-Zap70 in the subsets of both newly generated tTreg cells and newly generated tTcon cells of the naturally aged thymi (Fig 4A–4D) and in tTreg cells but not in tTcon cells from the FC-mOVA atrophied thymi infused with OT-II^+^ TCR-Tg pre-tTreg cells (Fig 4E). The increase of p-Zap70 in T clones indicates that the TCRs are activated and these thymocytes are activated to undergo either apoptosis or differentiation. Therefore, we investigated the apoptosis in tTreg cells and tTcon cells from the young and aged thymi of Rag-GFP reporter mice. This approach is used to either exclude a possibility that the increased tTreg cell proportion is due to aquiring a refractory behavior and subsequently resisting apoptosis or to confirm that the decreased tTcon cell proportion is due to increased apoptosis after TCR activation (increased p-Zap70). The results showed that in the atrophied thymus, apoptosis in newly generated tTreg cells was not reduced or increased compared to those in the normal thymus, whereas apoptosis in newly generated tTcon cells was increased (Fig 4F–4H). The results imply that increased TCR activation in tTreg cells leads to tTreg cell generation, while in tTcon cells it results in tTcon reduction in the aged thymus. Finally, we tested tTreg cell suppressive function to further comfirm that tTreg cells in the atrophied thymus do not possess intrinsic defects in function. This was done by comparing sorted, newly generated tTreg cells (Rag-GFP^+^CD4^SP^CD25^+^) from young WT, middle-aged WT, and middle-aged FC mice (S4A Fig). Due to CreER^T^-mediated autoleakage-related deletion of *FoxN1*^fx/fx^, mice in this group have a similar thymic profile to 18-month-old WT, which was shown in S1 Fig. We found that both normal and atrophied thymus-generated tTreg cells possessed similar suppressive function to T effectors (S4B Fig).

Taken together, the unchanged Treg generation in the atrophied thymus is relatively enhanced in activity, which is driven by the atrophied thymic microenvironment but unlikely to be due to age-related intrinsic changes in apoptosis or in loss of suppressive function of tTreg cells.

### Negative selection-related signals were decreased in CD4^SP^ thymocytes in both naturally aged and acute atrophied thymi

**Fig 5:**
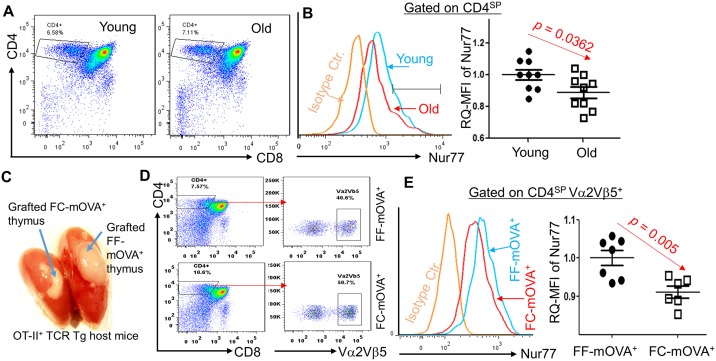
Thymic atrophy impaired negative selection signal Nur77 in CD4^SP^ thymocytes. Panels A and B: results from directly stained young and naturally aged thymocytes. **(A)** Flow cytometric gate strategy shows CD4^SP^ gate. **(B)** Left panel shows Nur77 in CD4^SP^ population; right panel is summarized RQ-MFI results of Nur77 levels in CD4^SP^ thymocytes of WT young and naturally aged mice. Panels C–E: results from grafted FF- and FC-mOVA thymi, which were grafted under the kidney capsule of OT-II^+^ TCR-Tg mice with *Rag*^−/−^ background (OT-II^+^/*Rag*^−/−^). **(C)** Image of grafted mOVA-Tg thymi under the kidney capsule of OT-II^+^/*Rag*^−/−^ mice, treated with TM x3. **(D)** A representative flow cytometric gate strategy shows Vα2^+^Vβ5^+^ thymocytes in CD4^SP^ population (these thymocytes were derived from OT-II^+^/*Rag*^−/−^ host mouse bone marrow progenitors) in the grafted FF- and FC-mOVA-Tg thymi. **(E)** Left panel: a representative histogram of Nur77 in the gates of CD4^SP^ and Vα2^+^Vβ5^+^ (primarily derived from OT-II^+^/*Rag*^−/−^ progenitors) thymocytes in the grafted FF- and FC-mOVA-Tg thymi; right panel: summarized results of RQ-MFI of Nur77 levels from the left panel. In panels B and E, a Student *t* test was used to determine statistical significance between groups, and all data are expressed as mean ± SEM. Each symbol represents an individual animal sample. Underlying data used in the generation of this figure (panels B and E) can be found in S1 Data. CD, cluster of differentiation; FC, *FoxN1*^fx/fx^/CreER^T^; FF, *FoxN1*^fx/fx^ without CreER^T^ for controls; *Foxn1*, Forkhead box protein N1; *FoxN1*^fx/fx^, *loxp*-flanked *FoxN1* gene; mOVA-Tg, membrane-bound ovalbumin transgenic mouse; Nur77, orphan nuclear receptor; OT-II, MHC class-II restricted ovalbumin-specific TCR transgenic mouse; RQ-MFI, relative quantitative Mean Fluorescence Intensity; WT, wild-type.

To determine the underlying mechanism leading to the relatively enhanced tTreg cell generation in the atrophied thymic microenvironment, we focused on TCR signaling strength, since it determines self-T clone fates. First, we looked at Nur77 as a representative negative selection marker gene, since insufficient Nur77 signaling leads to reduced clonal deletion [27] and enhanced tTreg cell generation [28]. We found that negative selection signaling molecule Nur77, as measured by the relative quantitative Mean Fluorescence Intensity (RQ-MFI) was reduced in either WT CD4^SP^ thymocytes of the naturally aged thymus (Fig 5A and 5B) or OT-II^+^ TCR-Tg CD4^SP^ thymocytes from the acute atrophied FC-mOVA thymus grafted under the kidney capsule of OT-II^+^ TCR-Tg recipients (Fig 5C shows an illustration of this setting; results are shown in Fig 5D and 5E).

**Fig 6:**
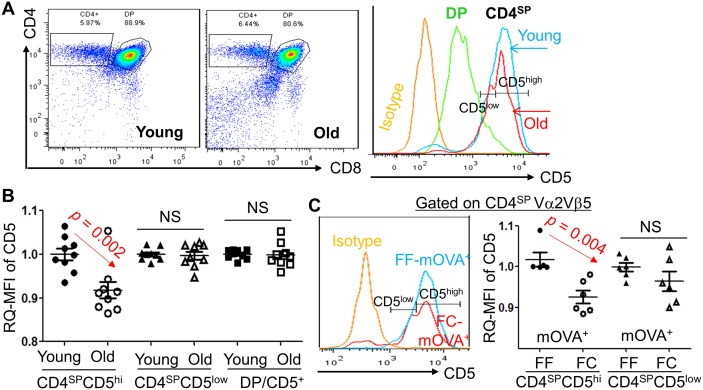
CD4^SP^CD5^hi^ thymocytes were decreased in either the naturally aged (≥ 18 months old) or the acute atrophied (*FoxN1* cKO) thymi. **(A)** A representative result from WT young and aged mice shows flow cytometric gate strategy for CD4^SP^, DP, and CD5^low^ and CD5^high^. **(B)** Summarized RQ-MFI of CD5 in various populations from panel A. **(C)** Results from grafted FF- and FC-mOVA-Tg thymi, which were under the kidney capsule of OT-II^+^/*Rag*^−/−^ host mice (Same setting as Fig 5C). Left panel: representative histograms of CD5^low^ and CD5^high^ in CD4^SP^ thymocytes (developed from seeded OT-II^+^/*Rag*^−/−^ bone marrow progenitors) in the grafted thymi of FF-mOVA-Tg (normal thymus, without CreER^T^) and FC-mOVA-Tg (atrophied thymus after TM x3) mice. Right panel: summarized results of RQ-MFI of CD5 from the left panel. A Student *t* test was used to determine statistical significance between groups, and all data are expressed as mean ± SEM. Each symbol represents an individual animal sample. Underlying data used in the generation of this figure (panels B and C) can be found in S1 Data. CD, cluster of differentiation; cKO, conditional knockout; CreER^T^, ubiquitous promoter-driven Cre-recombinase and estrogen-receptor fusion protein; DP, double positive; FC, *FoxN1*^fx/fx^/CreER^T^; FF, *FoxN1*^fx/fx^ without CreER^T^ for controls; *Foxn1*, Forkhead box protein N1; *FoxN1*^fx/fx^, *loxp*-flanked *FoxN1* gene; mOVA-Tg, membrane-bound ovalbumin transgenic mouse; OT-II, MHC class-II restricted ovalbumin-specific TCR transgenic mouse; RQ-MFI, relative quantitative Mean Fluorescence Intensity; WT, wild-type.

Although the role of CD5 in the thymus is for fine-tuning of TCR signaling, CD5 surface expression quantitatively correlates with TCR signal intensity [29], i.e., CD5^high^ expression in T cells is produced by a strong interaction between a high affinity/avidity self-pMHC to self-TCR [29–32], which triggers negative selection in the thymus [29]. CD5 expression is conversely related to tTreg cell selection, since insufficient CD5 in CD5-deficient mice causes an increase in tTreg cells [33]. We measured the CD4^SP^CD5^high^ thymocyte population either in the naturally aged thymus (Fig 6A and 6B) or in the acute atrophied FC-mOVA^+^ thymus under the kidney capsule of OT-II^+^ TCR-Tg mice, similar to the Fig 5C setting (Fig 6C). The MFI of CD5 in the CD4^SP^CD5^high^ population in both settings was decreased, but there was no difference in CD4^+^CD8^+^ double positive (DP) and CD4^SP^CD5^low^ populations (Fig 6B and 6C right panel).

These results implied insufficient negative selection signals were received by self-reactive TCRs in the atrophied thymus, which leads to a functional handicap of both negative selection and deletion of self-reactive T clones from the developing thymocyte pool, as we reported in our previous publications [13, 14].

### Capacity of presenting self-antigen on mTECs in the atrophied thymus declined

**Fig 7:**
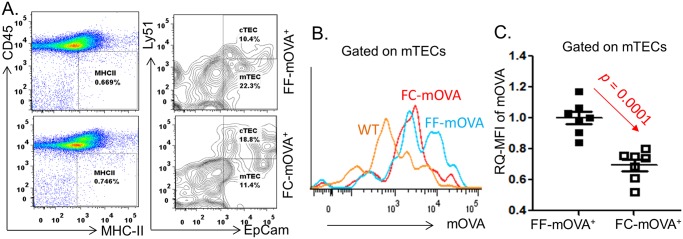
Capacity of mTEC self-antigen presentation was declined in the atrophied thymus demonstrated by a neo/mock self-antigen mOVA model. **(A)** A representative flow cytometric gate strategy shows CD45^-neg^MHC-II^+^ TECs (left panel) and Ly51^+^EpCam^+^ cTECs and Ly51^-neg^EpCam^+^ mTECs (right panel) from young 1.5-month-old FF thymus and 8-month-old FC (*FoxN1*^fx/fx^ with a CreER^T^ autoleakage) thymus. **(B)** A representative flow cytometric histogram shows expression of mOVA in mTECs of WT young (non-mOVA-Tg, orange), young 1.5-month-old FF-mOVA-Tg (blue), and 8-month-old FC-mOVA-Tg (red) mice. **(C)** A summary of mOVA expression in mTECs of young 1.5-month-old FF-mOVA-Tg thymus and 8-month-old FC-mOVA-Tg thymus. A Student *t* test was used to determine statistical significance between two groups, and in Panel C, the level bars are expressed as mean ± SEM. Each symbol represents an individual animal sample. Underlying data used in the generation of this figure can be found in S1 Data. CD, cluster of differentiation; cTEC, cortical thymic epithelial cells; FC, *FoxN1*^fx/fx^/CreER^T^; FF, *FoxN1*^fx/fx^ without CreER^T^ for controls; mTEC, medullary thymic epithelial cell; TEC, thymic epithelial cell; Tg, transgenic; mOVA-Tg, membrane-bound ovalbumin transgenic mouse.

TCR signaling strength is produced through an interaction between the self-pMHC presented on mTECs and the self-recognizing TCR expressed on self-T clones. Self-peptide presentation in mTECs is also termed “promiscuous expression” of peripheral tissue-specific antigens (TSAs), which distinguishes the TEC-autonomous gene expression. In addition, expression of most TSAs on mTECs is *Aire*-dependent. Given that aging primarily occurs in TECs [34] and the *Aire* gene is declined in mTECs on a per-cell basis in the atrophied thymus [14], this suggests that mTECs in the age-related atrophied thymus are unable to normally promiscuously express self-pMHC. Therefore, we hypothesized that the reduced TCR signaling strength is due to ineffective self-pMHC expression on the surface of these mTECs, leading to a reduction of interactive signaling to the self-T clones. This is unlikely to be a defect in TCR avidity since transfer of young thymocytes into the atrophied thymus showed the same relatively enhanced tendency of new tTreg cell generation (Fig 3). Therefore, we detected expression of a neo/mock-self-antigen (mOVA) on mTECs to mimic TSA expression in FF-mOVA and FC-mOVA mouse models. In this model, mOVA expression on mTECs is controlled by *Aire* gene [24], and the thymus of FC-mOVA experiences accelerated aging (resulting from a *FoxN1*^fx/fx^ deletion under a CreER^T^ autoleakage over time [12], see S1 Fig). Our results showed that mOVA expression in the FC-mOVA^+^ thymus was significantly reduced (Fig 7), which verified our hypothesis that mTECs in the atrophied thymus are unable to present self-antigen efficiently. This notion is also supported by our previous finding that *Aire*-dependent self-antigens (such as salivary protein-1, Insulin-1 and -2, intestinal fatty acid binding protein) were decreased in mTECs of the *FoxN1*-conditional knockout (cKO) atrophied thymus [13].

### The tTreg cell-required cytokines and NF-κB factor unlikely affected tTreg cell generation in the atrophied thymic microenvironment

To further identify the underlying mechanism by which thymic atrophy relatively enhances tTreg cell generation, we also considered that tTreg cell generation-required cytokines, such as interleukin-2 (IL-2) [35] and Transforming Growth Factor-Beta (TGFβ) [36, 37], as well as transcription factors, such as the Nuclear Factor kappa Beta (NF-κB) family [38, 39], which are primarily provided by the thymic microenvironment, could be altered in the atrophied thymus. We detected that in the naturally aged and acute atrophied FC thymi (S5 Fig), expression levels of IL-2 and TGFβ, as well as one of the NF-kB family of transcription factors, p105/p50, were unchanged compared to young and FF counterparts with normal thymi. Only expression of p105/p50 was slightly increased in the naturally aged thymus (S5 Fig, top panel), which was due to inflammation in the atrophied thymus. Although this increase could help tTreg cell maturation by inducing expression of forkhead box P3 (FoxP3) [38, 39], this is most likely not the primary mechanism for the enhancement of tTreg cell generation we observed in the atrophied thymus.

## Discussion

Using a combination of naturally aged and multiple genetically engineered mouse models and investigating signaling molecules in either the thymocyte or mTEC thymic cellular compartments, we have demonstrated that the aged, atrophied thymic microenvironment does not impair, but may relatively promote, tTreg cell generation. This evidence has revealed how central tolerance is established in the age-related atrophied thymic microenvironment, which we determined to be a heterogeneous effect of defective negative selection accompanied by relatively enhanced tTreg cell generation. This is probably a potential attempt by the atrophied thymus to desperately balance the defective negative selection by enhancing tTreg cell generation to maintain central T-cell tolerance. As we know, under normal conditions, two arms—namely, negative selection and tTreg cell generation—work in tandem for central T-cell tolerance establishment. However, in the age-related atrophied thymus, as one arm of tolerance induction (negative selection) fails, we assert that the second arm (tTreg cell generation) attempts to compensate, resulting in relative tTreg cell enhancement.

**Fig 8:**
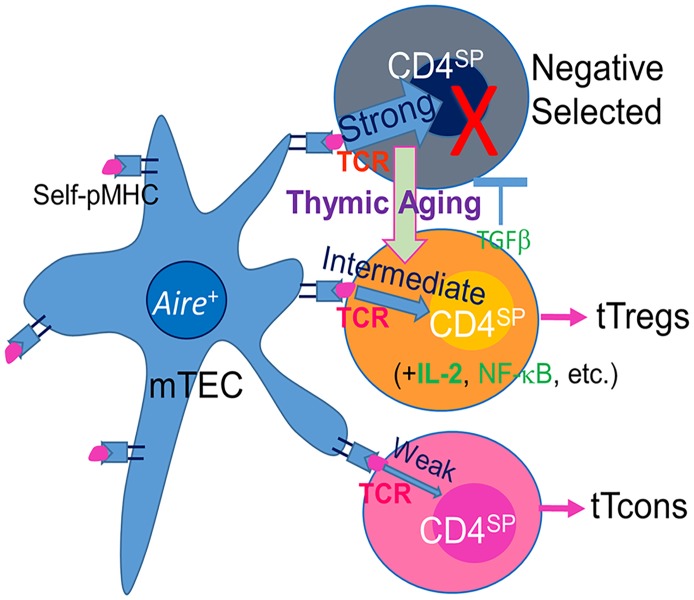
Schematic diagram of potential mechanism. Interaction between self-pMHC on mTEC and TCR on thymocytes produces three types of signaling strength to determine self-T clone fates. A strong signal leads to negative selection and results in the self-T clone depletion; an intermediate signal leads to thymocyte differentiation to a tTreg cell population; and a weak signal results in thymocyte survival to differentiate into tTcon cells. These signals can be detected by negative selection marker genes, such as Nur77 and CD5 expression on CD4^SP^ thymocytes. Thymic aging impairs mTECs, including a decline of *Aire* expression. It results in ineffective presentation of self-pMHC, thereby weakening the interactions between self-pMHC and TCR. Therefore, some self-T clones which should be depleted shift to tTreg cell generation due to strong signaling strength shifting toward intermediate signaling strength. CD, cluster of differentiation; mTEC, medullary thymic epithelial cell; self-pMHC, self-peptide-Major Histocompatibility Complex; TCR, T cell receptor; tTcon, thymic conventional T cell; tTreg, thymic regulatory T cell.

This mechanism can be explained by changes in TCR signaling strength in the thymus, which acts as a gate-keeper to determine thymocyte fate (diagrammed in Fig 8). Although this is the first time that this observation has been noted in the TEC-defective and age-related atrophied thymus, the phenomenon has been demonstrated by several loss-of-function young mouse models. The TCR signaling is produced by interaction between the two parties: mTECs and thymocytes. The first established mouse model for studying this signaling pathway contains an alteration in the thymocyte side. As we know, when the TCR responds to antigen recognition, the immunoreceptor tyrosine-based activation motifs (ITAMs) are activated and the signaling kinase Zap70 is subsequently phosphorylated. A mouse model with a knock-in allele of TCRξ chain gene with tyrosine-to-phenylalanine mutations in 6 out of 10 ITAMs leads to a 60% decrease in TCR signaling potential [40]. This mouse model exhibited a defect in negative selection but an increase in tTreg cell generation [40]. The second mouse model for the TCR signaling pathway contains a change in the TEC compartment. Tg expression of a microRNA targeting Major Histocompatibility Complex (MHC) class-II transactivator (CIITA) results in declined MHC-II on mTECs [26], which leads to an insufficiency for mTECs to present self-antigens for normal interaction with TCR. This also resulted in an enhancement of tTreg cell generation at the expense of negative selection [26]. These two models from both parties of the signaling interaction support one conclusion, namely, that shifting a strong signal to an intermediate signal due to any weakening of the interaction between self-pMHC and self-reactive TCR allows the self-reactive thymocytes, which should be deleted, to differentiate to tTreg cells [25]. In the age-related atrophied thymus, the capacity of self-antigen presentation (promiscuous expression of TSAs) on mTECs is reduced due to a defect in TEC homeostasis [12, 34] associated with a decline of *Aire* gene expression [13, 14]. Although there has been controversy about the role of *Aire* gene in positive selection of Treg cells [41], it has been accepted that *Aire* regulates *Aire*-dependent self-peptide expression on mTECs. Moreover, total [42] or certain specific [43] tTreg cells were found not to be decreased in *Aire* deficient models. Therefore, the interaction of self-pMHC and self-reactive TCR produces weak signals in the age-related atrophied thymus, which results in a similar outcome as illustrated by the second model: ineffective mTECs [26].

Our results presented here are inconsistent with a published report in which the phenotype of “substantially decreased new tTreg cells and augmented recirculating pTreg cells among thymic Treg cells with age” was observed [17]. These phenotypes were based on different observations by comparing Rag-GFP^+^ Treg cells versus Rag-GFP^-neg^ Treg cells within a thymic Treg subset and Rag-GFP^+^ Tcon cells versus Rag-GFP^-neg^ Tcon cells within a thymic Tcon subset, rather than comparing Treg cells to Tcon cells within the Rag-GFP^+^ CD4^SP^ or Rag-GFP^-neg^ CD4^SP^ subsets. The results presented a proportion change between newly generated (Rag-GFP^+^) and reentered (Rag-GFP^-neg^) portions only in the same subset, rather than reflecting the changes in an integrated total CD4^SP^ population. Therefore, it does not exhibit integrated changes in newly generated and reentered Treg cells compared to their counterpart Tcon cells and within the entire CD4^SP^ cell population. Regarding inhibition of the de novo tTreg cell generation by reentered pTreg cells in the thymus, these cells may be importantly involved in a potential negative feedback loop of regulation [17]. Since pTreg cells are increased in aged mice and humans due to declined *Bim* gene-resulted accumulation [15], it is a concern that aged pTreg cells may reenter the aged thymus more than their young counterparts to augment inhibition of new tTreg cell generation. However, we did not see this, since there was no particularly different tendency of re-entry of young pTreg cells (FC model possesses a young periphery with no accumulated Treg cells and no decreased *Bim* gene [14]) or old pTreg cells (naturally aged model) in the atrophied thymus (Figs 1D and 2C). Therefore, the pTreg reentering mechanism should be the same in either young or aged individuals.

Development of tTreg cells in the thymus is primarily dependent on thymic microenvironments. We found that the atrophied thymic microenvironment favors tTreg cell development because they demonstrated more activity compared to their counterparts in the normal thymus (Figs 3 and 4A–4E) and compared to tTcon cells in the same atrophied thymus (Fig 4E). This is not due to apoptotic resistance by a refractory feature (Fig 4F–4H). Except for tTreg cells, apoptosis in tTcon cells (Fig 4F–4H) and in other populations of FC mice [18] is increased. Increased apoptotic microenvironment was reported to drive tTreg cell generation through the apoptosis-TGFβ-FoxP3 axis [21]. Although we did not find any increase of TGFβ (S5 Fig), we believe that increased apoptosis in the thymus induces an increased pro-inflammatory condition that will be favorable for tTreg cell development [38, 39].

It is well known that in aged humans and mice, an increased pTreg pool has been observed not only in proportion and absolute Treg numbers [15, 44] but also in function [16], which negatively affects anti-infection [45] and antitumor [46] immunity. Given the fact that tTreg cell generation is relatively enhanced in the aged atrophied thymus, we have a reason to deduce that output of tTreg cells may impact the pTreg pool in aged individuals. Although evidence is still insufficient, it was reported that in the pTreg pool, the proportion of natural Treg cells (i.e., tTreg cells, based on recommended Treg nomenclature [47]) versus inducible Treg cells is increased with age [48]. This not only implies that older individuals are less capable of generating inducible Treg cells [48] due to an intrinsic defect in these extrathymically induced Treg cells [49] but also indirectly suggests that aged thymus-derived tTreg cells are increased in the peripheral pTreg pool. Our peripheral data also provide evidence that recent thymic emigrant (Rag-GFP^+^) Treg cells in the spleens of aged mice were increased, while Rag-GFP^+^ CD4^SP^ Tcon cells in the spleens of aged mice were decreased (S6 Fig). Another profound question is why this increased tTreg cell involvement in the aged pTreg pool cannot suppress self-reactivity-induced inflammaging [14], although it suppresses normal anti-infection and antitumor immunity [45, 46]. This can probably be accounted for because reduced capacity of presenting self-pMHC diverts the lineage of some tTreg cell-biased clones into self-reactive tTcon cells [50]. These tTcon cells are released to the periphery to elevate self-reactivity, which is our next in-depth work. There is also another possibility of an imbalance between increased total Treg cells and decreased specific Treg cells, i.e., a biased tTreg cell specificity, which is seen in unpublished studies of certain autoimmune diseases and will be substantially addressed in our future work as well. However, as we have reported here, the atrophied thymus indeed attempts to balance the defective negative selection by enhancing tTreg cell generation to try to maintain central T-cell immune tolerance in the elderly. This mechanism should contribute to an optimal balance in most aged people since most of them do not have an onset of age-related autoimmune diseases, despite most elderly people having increased chronic inflammation. However, once this balance is broken, age-related inflammatory diseases could take place.

## Materials and methods

### Ethics statement

All experiments were conducted on C57BL/6 genetic background mice, and the performance was in compliance with a protocol (IACUC-2016-0037) approved by the Institutional Animal Care and Use Committee (IACUC) of the University of North Texas Health Science Center, in accordance with guidelines on animal welfare of the National Institutes of Health. All efforts were taken to minimize mouse usage to maximize necessary results; provide the best veterinary care; and minimize discomfort, distress, and surgery with anesthetic procedures and euthanasia.

### Mice, crossbreeding, and animal care

Various genetically manipulated mouse colonies (all on C57BL/6 genetic background) and their crossbreeding schemes have been described in our previous publication [14]. Briefly, *FoxN1*^fx/fx^ [18] mice were crossbred with CMV-promoter-driven CreER^T^ (ubiquitous promoter-driven Cre-recombinase and estrogen-receptor fusion protein) Tg mice, then with *rag*-*gfp* reporter mice. The *FoxN1*^fx/fx^ cKO was through either a TM-induced acute deletion or CreER^T^ autoleakage-mediated time-course deletion [12]. Both deletions induce accelerated thymic atrophy. We term this model the FC mouse and FF mouse. The thymocyte profiles in a 1-month-old FC thymus without TM treatment is exactly the same as those in 1-month-old WT mice, whereas the thymocyte profiles in a young FC mouse treated with TM (1 mg/10 g body weight/day) for 3 consecutive days (TM x3) and FC mouse is housed for 6 months without any TM treatment with a CreER^T^ age-related autoleakage are the same as in the naturally aged WT (≥18-month-old) thymus (details in S1 Fig). RIP-mOVA Tg mice (#005431) and OT-II^+^ TCR Tg mice (#004194) were purchased from Jackson Lab. OT-II^+^ TCR Tg mice were cross-bred with *Rag*^−/−^ mice (Jackson Lab #002216). These mice were termed OT-II^+^/Rag ^−/−^ mice (see S3 Fig). Aged WT mice were ordered from the National Institute on Aging, aged rodent colonies. Mouse ages are indicated in each figure legend or defined young (1–2 months old) and naturally aged (in most situations, they were ≥ 18 months old).

### Intrathymic transfer of pre-tTreg cells

CD8^SP^ and CD8^+^CD4^+^ DP OT-II^+^ TCR-Tg thymocytes were depleted through an anti-CD8 cytotoxic antibody (clone HO-2.2) and Low-Toxic-M complement. Then, the live thymocytes were sorted by flow cytometry for pre-tTreg cells (CD4^SP^ and CD25^-neg^ thymocytes). The sorted pre-tTreg cells were i.t. injected into the thymus (details in our previous publication [51]) of young FF- or FC-mOVA-Tg mice (1 × 10^6^ cells per recipient mouse), respectively. Five days after the pre-tTreg cell injection, the thymocytes with Vα2^+^V***β***5^+^ TCR chain types (mostly OT-II^+^ TCR-Tg thymocytes) in recipient FF- or FC-mOVA thymi were collected for analysis of newly generated tTreg cells (CD4^SP^CD25^+^FoxP3^+^) versus tTcon cells (CD4^SP^CD25^-neg^FoxP3^-neg^). Meanwhile, recipient mice were intraperitoneally (i.p.) injected with TM (1 mg/10 g body weight/day) for 3 consecutive days (TM x3): one day before, one day along with i.t. injection, and one day after to induce thymic atrophy in FC-mOVA mice (detailed procedure is outlined in Fig 3A).

### Kidney capsule transplantation of thymic lobes

The surgical operation of the kidney capsule transplantation was performed as previously described [14]. Briefly, intact thymic lobes from FF- or FC-mOVA-Tg newborn mice were directly transplanted into young host OT-II^+^ TCR-Tg mice under the kidney capsule (a diagram is shown in Fig 5C). One week after the transplantation, the host mice were i.p. injected with TM x3 to induce deletion of the *FoxN1*^fx/fx^ gene in the grafted thymic lobes. Two weeks after the last TM injection, the grafted thymi were isolated for flow cytometric analysis (FACS) of Nur77 and CD5 in CD4^SP^ cells carrying Vα2^+^V***β***5^+^ TCR chain type for detecting cells bearing OT-II^+^ TCR-Tg T cells.

### Flow cytometric assay of thymocytes and TECs

For thymocyte staining, single-thymocyte suspensions were prepared from the thymi of mice using a 70-μm cell strainer. Samples were then stained with specific fluorochrome-conjugated antibodies of cell surface CD markers, indicated in each figure legend, and then fixed and permeabilized with fixation/permeabilization buffer (eBioscience Treg staining kit, #88-8115-40), per company’s instruction, followed by intracellular staining for PE-anti-FoxP3 (eBioscience Cat# 12-5773-82) or FITC-anti-FoxP3 (eBioscience Cat# 11-5773-82), and/or PE-anti-p-Zap70 (Cell Signaling, Cat# 14791, clone 65E4, recognizing phosphorylation at Tyr319/Syk [Tyr352]), PE-anti-Nur77 (eBioscience, Cat# 12-5965-82, clone 12.14), and AlexaFluor488-anti-GFP (BioLegend, Cat# FM264G). For TEC staining, the thymus was cut into pieces, then was digested with Collagenase-V/DNase-I, as per previously published methods [51], then stained with surface molecules: fluorochrome-conjugated anti-CD45 (clone 30-F11), -MHC-II (clone M5/114.15.2), -Ly51 (clone 6C3), and -EpCAM (clone G8.8), which were purchased from BioLegend, and FITC-anti-Ovalbumin from AbCam (Cat# ab85584). Flow cytometry was performed using an LSRII flow cytometer (BD Biosciences) with 6 colors, and data were analyzed using Flow-Jo software.

### Annexin-V-based apoptosis assays of tTreg cells

Thymocytes were harvested from young (2 months old) and aged (16 months old) Rag-GFP reporter mice, and stained with surface CD markers as described above. Then, the cells were washed with Annexin-V binding buffer and incubated in APC-Annexin-V (BioLegend, Cat# 640920) at a 1:20 dilution with Annexin-V binding buffer (10 mM Hepes adjusted to pH 7.4, 140 mM NaCl and 2.5 mM CaCl_2_) for 15 min at room temperature in the dark. The cells were then washed with PBS and fixed and permeabilized with fixation/permeabilization buffer, followed by eBioScience intracellular staining protocol for FoxP3 and GFP. Positive control cells were aliquoted from the thymocytes and made by incubating at 55°C for 20 min to induce cell death before staining.

### Suppressive function assay of tTreg cells

Antigen presenting cells (APCs) were prepared from young WT mouse bone marrow-derived dendritic cells (with rGM-CSF and rIL-4 induction to get 85% CD11c^+^ cells) and were treated with irradiation of 2,000 Rads. T effector (Teff) cells were prepared from young WT spleen through direct flow cytometric sorting of CD4^+^CD25^-neg^ spleen T cells; details were outlined in our and others’ previous publications [14, 16]. The tested newly generated tTreg cells (CD4^SP^CD25^+^Rag-GFP^+^) were sorted from 3 groups of thymocytes: young (approximately 6–8 weeks old) Rag-GFP WT; middle-aged (approximately 12 months old) Rag-GFP WT; and middle-aged (≥ 8 months old) FC-Rag-GFP mice (similar to ≥18-month-old WT with the atrophied thymus, See S1 Fig). The sorting purity check showed 95%–99% (S4A Fig). A mixed culture with APCs (5 × 10^3^/well), Teff cells (5 × 10^4^/well), and tTreg cells (2.5 × 10^4^/well) supplied with CD3ε (1 μg/ml) and CD28 (2 μg/ml) antibodies were set in 96-well U-bottom plates for 72 hours. Proliferation of cells was determined using CellTiter 96 Aqueous One Solution Reagent (Promega, Cat#TB245) following the company’s protocol: adding 20 μl of solution per well for additional 2-hour culture, and absorbance was measured at 490 nm using an ELISA 96-well plate reader (BioTek ELx800).

### Real-time RT-PCR for cytokine expression assay

Total RNAs were isolated with TRIzol reagent and reverse transcribed (RT) to cDNA with the SuperScriptIII cDNA kit (Invitrogen/ThermoFisher Scientific). Real-time RT-PCR was performed with TaqMan reagents and primers (TGFβ, NFκBs, IL-2, and housekeeping GAPDH and 18sRNA; the primers and probes were purchased from Invitrogen) [18]. The relative expression levels of mRNAs from the thymi were internally normalized to both GAPDH and 18sRNA levels, then compared to a ΔΔC_T_ value from pooled young samples, which was always arbitrarily set as 1.0 in each real-time PCR reaction.

### Statistics

For evaluation of group differences, the unpaired two-tailed Student *t* test was used, assuming equal variance. Differences were considered statistically significant at values of * *p* < 0.05; ** *p* < 0.01; and *** *p* < 0.001. Curves in Figs 1 and 2 and S2 Fig are nonlinear one-phase decay assay and linear regression assay, as indicated in the figure legends. All statistics were analyzed with Prism software (GraphPad).

## Supporting information

S1 Fig The thymus in *FoxN1* conditional knockout mice shows a similar profile to the thymus of WT naturally aged mice. (**A**) Flow cytometric profile of CD4 versus CD8 from the 5 groups of various mice. (**B**) Total thymocyte number in four thymocyte subsets of 5 mouse groups. (**C**) Proportions of thymocytes in four thymocyte subsets of 5 mouse groups. Mouse numbers in each group are 5–15 animals. The thymus of *FoxN1*^fx/fx^-CreER^T^ (FC)-Young (1 month old) was not treated with tamoxifen (TM), showed the same profile as WT-young; while treated with TM 3 times (TM x3), showed the same profile as WT-aged (18 Months old) thymus. FC-6-month-old thymus without treatment with TM (with CreER^T^-mediated auto-leaky deletion of *FoxN1*^fx/fx^), also showed the same profile as WT-aged (18 Months old) thymus. NS = not significant between groups; SDs = significant differences (either *p* < 0.05 or *p* < 0.01) between any of these groups (ANOVA analysis). The results suggest that WT-young and FC-young (without TM treatment) have the same profile, while WT-aged (18 Months old), FC-young (TM x3), and FC-6 Months old (without TM treatment) possess the same profile. Underlying data used in the generation of this figure can be found in S1 Data. CD, cluster of differentiation; CreER^T^, ubiquitous promoter-driven Cre-recombinase and estrogen-receptor fusion protein; FC, *FoxN1*^fx/fx^/CreER^T^; FF, *FoxN1*^fx/fx^ without CreER^T^ for controls; *Foxn1*, Forkhead box protein N1; *FoxN1*^fx/fx^, *loxp*-flanked *FoxN1* gene; mOVA-Tg, membrane-bound ovalbumin transgenic mouse; TM, tamoxifen; WT, wild-type.
(TIF)

S2 Fig Corresponding to Fig 1C right panel (A) and Fig 2B right panel (B), and comparison of absolute cell numbers of thymic Treg cells and Tcon cells at the age of 4 weeks versus 55 weeks from Figs 1C and 2B. (**A** and **B**) The curves are nonlinear one-phase decay; the results demonstrated that absolute cell numbers of tTreg cells were not reduced with age, while the numbers of tTcon cells were dramatically reduced with age. (**C**) Summarized results of absolute cell numbers of tTcon and tTreg at the ages of 4 weeks and 55 weeks from the *FoxN1*^fx/fx^*-*CreER^T^ (autoleakage-induced deletion with time) and naturally aged thymus, respectively, from which the tTreg cells were not different between the two age groups, while tTcon cells were significantly reduced in the aged group compared to young groups. SD = Standard Deviation; NS = Not Significant. Underlying data used in the generation of panels A and B can be found in S1 Data. CreER^T^, ubiquitous promoter-driven Cre-recombinase and estrogen-receptor fusion protein; *FoxN1*^fx/fx^, *loxp*-flanked *FoxN1* gene; Tcon, conventional T cell; tTcon, thymic conventional T cell; tTreg, thymic regulatory T cell.
(TIF)

S3 Fig The generation of OT-II TCR-Tg mice with *RAG*-knockout (*Rag*^−/−^) background. (**A**) Comparison of Vα2 and Vβ5 double TCR positive CD4^SP^ thymocytes in WT, OT-II TCR-Tg only, *Rag*^−/−^ only, and OT-II TCR-Tg with *Rag*^−/−^ background mice; (**B**) Comparison of Vα2 and Vβ5 double TCR positive CD3^+^CD4^+^ splenic cells in four genotypic mice. The results indicated that we successfully generated OT-II TCR-Tg with *Rag*^−/−^ background mice, in which Vα2Vβ5 TCR^+^ CD4^SP^ population is dramatically increased, while CD8^SP^ and B cells are dramatically decreased. CD, cluster of differentiation; OT-II^+^ TCR Tg, MHC class-II restricted ovalbumin-specific TCR transgenic; RAG, Recombination activating gene; TCR, T cell receptor; Tg, transgenic; WT, wild-type.
(TIF)

S4 Fig Normal and atrophied thymus-generated tTreg cells possess the similar suppressive function. Sorted newly generated (Rag-GFP^+^) tTreg (CD4^+^CD25^+^) cells from young WT, middle-aged WT, and middle-aged FC (with CreER^T^-mediated auto-leaky deletion of *FoxN1*^fx/fx^, thymocyte profile is similar to aged WT—See S1 Fig) mice were cultured with APCs (bone marrow-derived DCs), supplied with anti-CD3ε (1μg/ml) and anti-CD28 (2μg/ml), with or without Teff cells (CD4^+^CD25^-^) sorted from young WT splenic cells. Cells were cultured for 3 days, then CellTiter 96 AQ reagent (Promega) was added for 2–4 hours of culture, then reaction was analyzed by Absorber Reader at 490 nm. (**A**) Results of tTreg cell sorting purity test; (**B**) Summarized results of tTreg cell suppressive capacity from the tTreg cell sorting of three times with at least four animals in each group. The results suggest that there is no difference of tTreg cell suppressive capacity among young, middle-aged, and middle-aged FC (similar to naturally old, i.e., ≥ 18-month-old) mice. Underlying data used in the generation of this figure (panel B) can be found in S1 Data. CD, cluster of differentiation; CreER^T^, ubiquitous promoter-driven Cre-recombinase and estrogen-receptor fusion protein; FC, *FoxN1*^fx/fx^/CreER^T^; *FoxN1*, Forkhead box protein N1; *FoxN1*^fx/fx^ or *FoxN1-*floxed, *loxp*-flanked *FoxN1* gene; GFP, green fluorescent protein; Teff, T effector cell; tTreg, thymic regulatory T cell; WT, wild-type.
(TIF)

S5 Fig Relative expression of tTreg cell generation-required cytokines and a transcription factor (NF-κB1) in the normal and atrophied thymi with real-time RT-PCR. (**Top**) The mRNAs from the thymi of WT young and naturally aged mice; (**Bottom**) The mRNAs from thymi of FF (*FoxN1*^fx/fx^, without uCreER^T^) and FC (*FoxN1*^fx/fx^ with CreER^T^, conditional knockout) mice (both groups treated with TM x3). A TaqMan-based real-time RT-PCR was conducted with TaqMan primers and probes to TGFβ, NF-κB (p105/p50), and IL-2, along with house-keeping genes, GAPDH and 18sRNA, for normalization. A Student *t*-test was used to determine statistical significance between groups. Data are expressed as mean ± SEM. Each symbol represents an animal. Underlying data used in the generation of this figure can be found in S1 Data. CreER^T^, ubiquitous promoter-driven Cre-recombinase and estrogen-receptor fusion protein; FC, *FoxN1*^fx/fx^/CreER^T^; *FoxN1*, Forkhead box protein N1; *FoxN1*^fx/fx^, *loxp*-flanked *FoxN1* gene; IL-2, interleukin-2; NF-κB, Nuclear Factor kappa Beta; RT, reverse transcribed; PCR, polymerase chain reaction; tTreg, thymic regulatory T cell.
(TIF)

S6 Fig RTE Treg cells were increased and RTE Tcon cells were decreased in the spleen of aged Rag-GFP reporter mice. (**A**) Flow cytometric gate strategy shows gates of splenic Treg cells and Tcon cells from isotype control sample (mixture of young and old spleen cells stained with isotype control antibody for FoxP3); Young and old Rag-GFP reporter mice. (**B**) A summary of percentages of splenic Tcon cells and Treg cells in young and old mice, showing decreased RTE Tcon cells and increased RTE Treg cells in the old spleens. Underlying data used in the generation of this figure can be found in S1 Data. FoxP3, forkhead box P3; GFP, green fluorescent protein; RTE, recent thymic emigrant; Tcon, conventional T cell; Treg, regulatory T cell; tTcon, thymic conventional T cell; tTreg, thymic regulatory T cell.
(TIF)

S1 Data All individual numerical values which underlie the summary data shown in the following figures: Figs 1B, 1C, 1D, 2B, 2C, 3C, 3D, 4D, 4E, 4H, 5B, 5E, 6B, 6C and 7C; S1B, S1C, S2A, S2B, S4B, S5 and S6B Figs. (XLSX)

## Abbreviations

APCs: antigen presenting cells
CD: cluster of differentiation
CIITA: class-II transactivator
cKO: conditional knockout
CreERT: ubiquitous promoter-driven Cre-recombinase and estrogen-receptor fusion protein
DP: double positive
FACS: flow cytometric analysis
FC: *FoxN1*^fx/fx^/CreER^T^
FF: *FoxN1*^fx/fx^ without CreER^T^ for controls
*FoxN1*: Forkhead box protein N1
FoxP3: forkhead box P3
GFP: green fluorescent protein
i.t.: intrathymically
IL-2: interleukin-2
ITAMs: immunoreceptor tyrosine-based activation motifs
MFI: mean fluorescent intensity
MHC: Major Histocompatibility Complex
mOVA: membrane-bound ovalbumin
mTEC: medullary thymic epithelial cell
NF-κB: Nuclear Factor kappa Beta
pTreg: peripheral regulatory T cell
p-Zap70: phosphoralated-Zap70
*Rag*: recombination-activating gene
Rag-GFP^-neg^: Rag-GFP reporter negative
RIP: rat insulin promoter
RQ-MFI: relative quantitative Mean Fluorescence Intensity
RT: reverse transcribed
self-pMHC: self-peptide-Major Histocompatibility Complex
SP: single positive
Tcon: conventional T cell
TCR: T cell receptor
TEC: thymic epithelial cell
Teff: T effector cells
Tg: transgenic
TGFβ: Transforming Growth Factor-Beta
TM: tamoxifen
Treg: regulatory T cell
TSA: tissue-specific antigen
tTcon: thymic conventional T cell
tTreg: thymic regulatory T cell
WT: wild-type
Zap70: Zeta-chain-associated protein kinase 70
