# Supplementary figures and images for: Capacity of tTreg generation is not impaired in the atrophied thymus
Source: PLoS Biol. 2017 Nov 8;15(11):e2003352. doi: 10.1371/journal.pbio.2003352 (PMC5695848; doi:10.1371/journal.pbio.2003352)

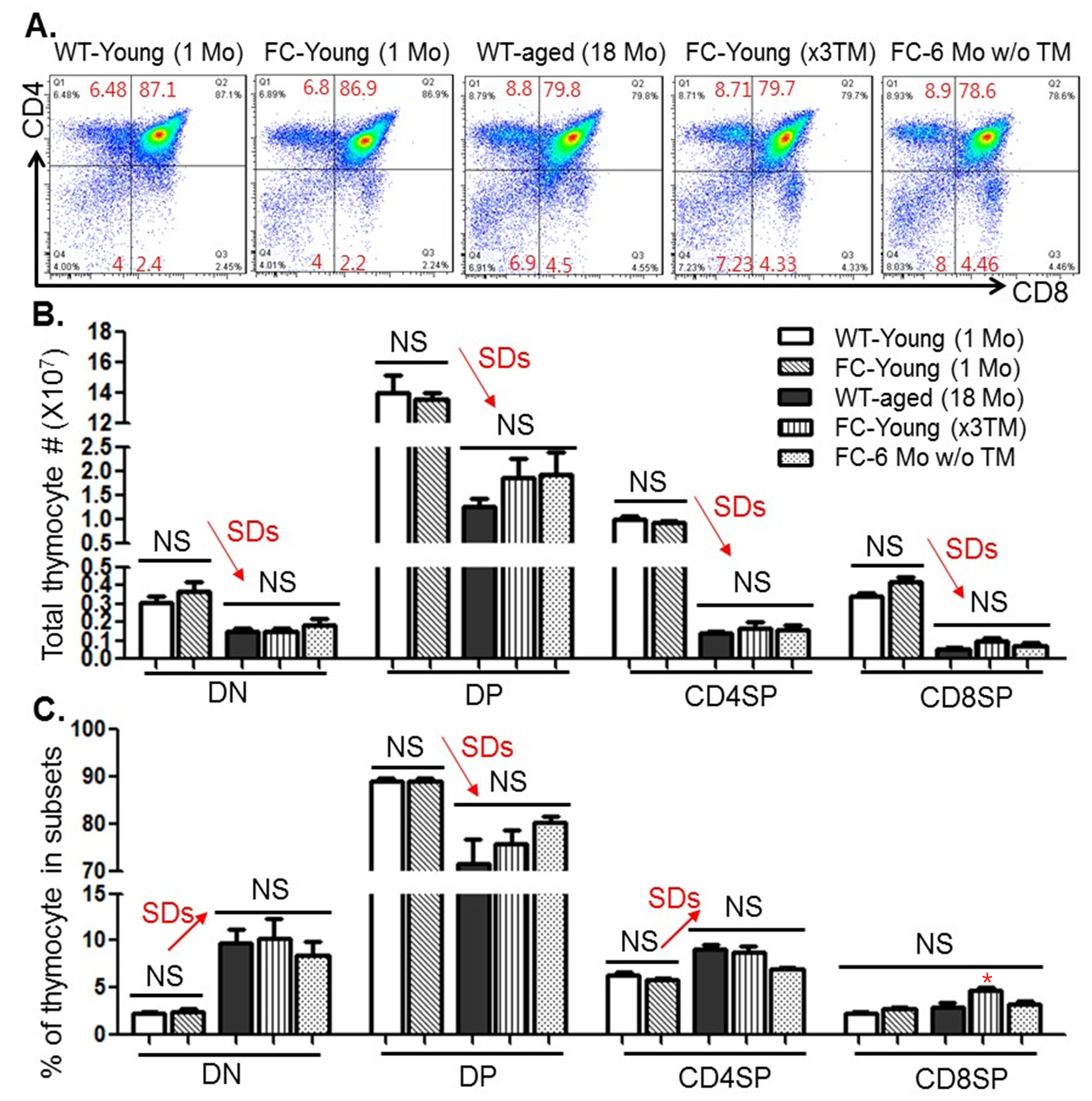

Supplement: S1 Fig — (A) Flow cytometric profile of CD4 versus CD8 from the 5 groups of various mice. (B) Total thymocyte number in four thymocyte subsets of 5 mouse groups. (C) Proportions of thymocytes in four thymocyte subsets of 5 mouse groups. Mouse numbers in each group are 5–15 animals. The thymus of FoxN1fx/fx-CreERT (FC)-Young (1 month old) was not treated with tamoxifen (TM), showed the same profile as WT-young; while treated with TM 3 times (TM x3), showed the same profile as WT-aged (18 Months old) thymus. FC-6-month-old thymus without treatment with TM (with CreERT-mediated auto-leaky deletion of FoxN1fx/fx), also showed the same profile as WT-aged (18 Months old) thymus. NS = not significant between groups; SDs = significant differences (either p < 0.05 or p < 0.01) between any of these groups (ANOVA analysis). The results suggest that WT-young and FC-young (without TM treatment) have the same profile, while WT-aged (18 Months old), FC-young (TM x3), and FC-6 Months old (without TM treatment) possess the same profile. Underlying data used in the generation of this figure can be found in S1 Data. CD, cluster of differentiation; CreERT, ubiquitous promoter-driven Cre-recombinase and estrogen-receptor fusion protein; FC, FoxN1fx/fx/CreERT; FF, FoxN1fx/fx without CreERT for controls; Foxn1, Forkhead box protein N1; FoxN1fx/fx, loxp-flanked FoxN1 gene; mOVA-Tg, membrane-bound ovalbumin transgenic mouse; TM, tamoxifen; WT, wild-type. (TIF) [file pbio.2003352.s001.tif]

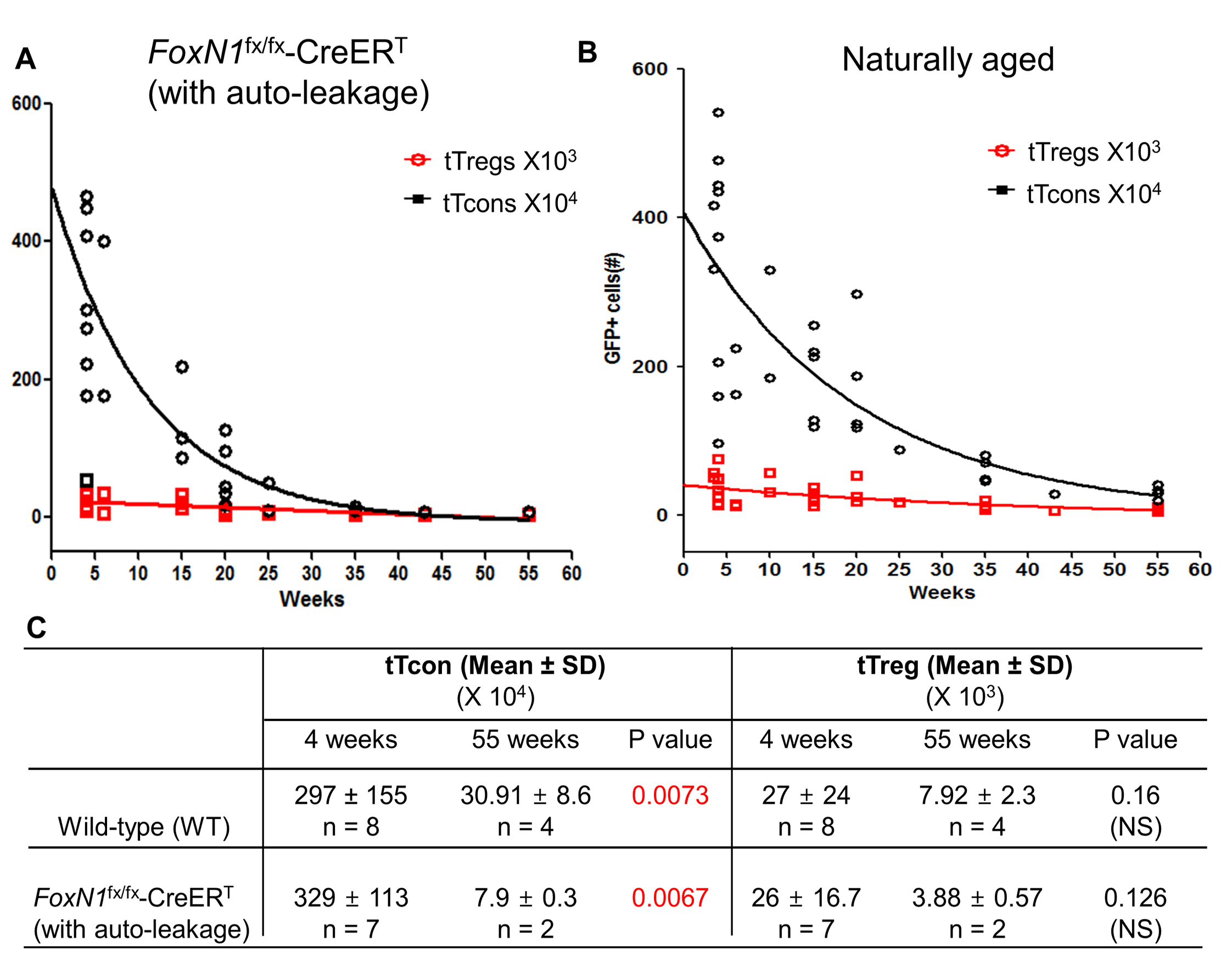

Supplement: S2 Fig — (A and B) The curves are nonlinear one-phase decay; the results demonstrated that absolute cell numbers of tTreg cells were not reduced with age, while the numbers of tTcon cells were dramatically reduced with age. (C) Summarized results of absolute cell numbers of tTcon and tTreg at the ages of 4 weeks and 55 weeks from the FoxN1fx/fx-CreERT (autoleakage-induced deletion with time) and naturally aged thymus, respectively, from which the tTreg cells were not different between the two age groups, while tTcon cells were significantly reduced in the aged group compared to young groups. SD = Standard Deviation; NS = Not Significant. Underlying data used in the generation of panels A and B can be found in S1 Data. CreERT, ubiquitous promoter-driven Cre-recombinase and estrogen-receptor fusion protein; FoxN1fx/fx, loxp-flanked FoxN1 gene; Tcon, conventional T cell; tTcon, thymic conventional T cell; tTreg, thymic regulatory T cell. (TIF) [file pbio.2003352.s002.tif]

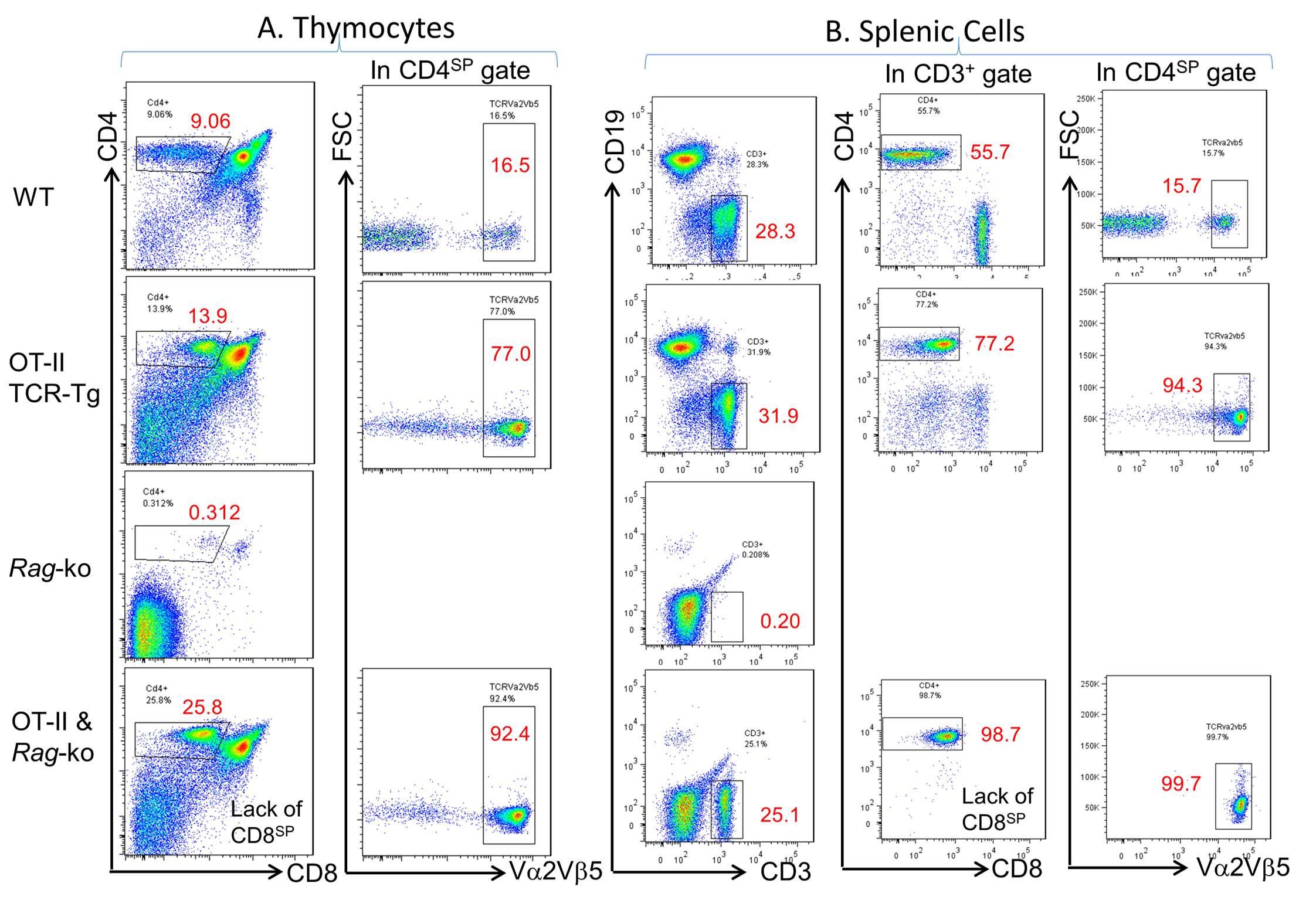

Supplement: S3 Fig — (A) Comparison of Vα2 and Vβ5 double TCR positive CD4SP thymocytes in WT, OT-II TCR-Tg only, Rag−/− only, and OT-II TCR-Tg with Rag−/− background mice; (B) Comparison of Vα2 and Vβ5 double TCR positive CD3+CD4+ splenic cells in four genotypic mice. The results indicated that we successfully generated OT-II TCR-Tg with Rag−/− background mice, in which Vα2Vβ5 TCR+ CD4SP population is dramatically increased, while CD8SP and B cells are dramatically decreased. CD, cluster of differentiation; OT-II+ TCR Tg, MHC class-II restricted ovalbumin-specific TCR transgenic; RAG, Recombination activating gene; TCR, T cell receptor; Tg, transgenic; WT, wild-type. (TIF) [file pbio.2003352.s003.tif]

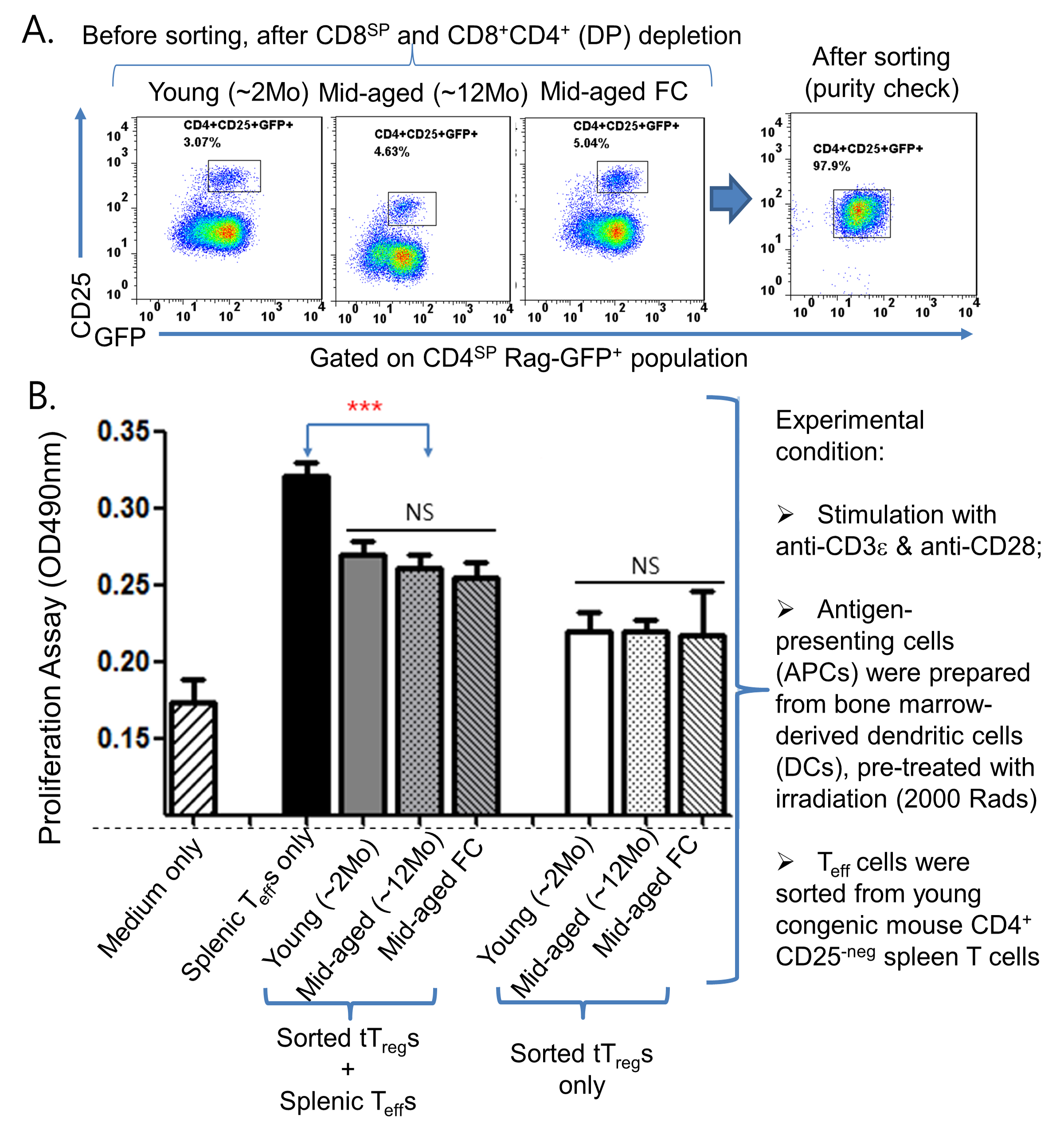

Supplement: S4 Fig — Sorted newly generated (Rag-GFP+) tTreg (CD4+CD25+) cells from young WT, middle-aged WT, and middle-aged FC (with CreERT-mediated auto-leaky deletion of FoxN1fx/fx, thymocyte profile is similar to aged WT—See S1 Fig) mice were cultured with APCs (bone marrow-derived DCs), supplied with anti-CD3ε (1μg/ml) and anti-CD28 (2μg/ml), with or without Teff cells (CD4+CD25-) sorted from young WT splenic cells. Cells were cultured for 3 days, then CellTiter 96 AQ reagent (Promega) was added for 2–4 hours of culture, then reaction was analyzed by Absorber Reader at 490 nm. (A) Results of tTreg cell sorting purity test; (B) Summarized results of tTreg cell suppressive capacity from the tTreg cell sorting of three times with at least four animals in each group. The results suggest that there is no difference of tTreg cell suppressive capacity among young, middle-aged, and middle-aged FC (similar to naturally old, i.e., ≥ 18-month-old) mice. Underlying data used in the generation of this figure (panel B) can be found in S1 Data. CD, cluster of differentiation; CreERT, ubiquitous promoter-driven Cre-recombinase and estrogen-receptor fusion protein; FC, FoxN1fx/fx/CreERT; FoxN1, Forkhead box protein N1; FoxN1fx/fx or FoxN1-floxed, loxp-flanked FoxN1 gene; GFP, green fluorescent protein; Teff, T effector cell; tTreg, thymic regulatory T cell; WT, wild-type. (TIF) [file pbio.2003352.s004.tif]

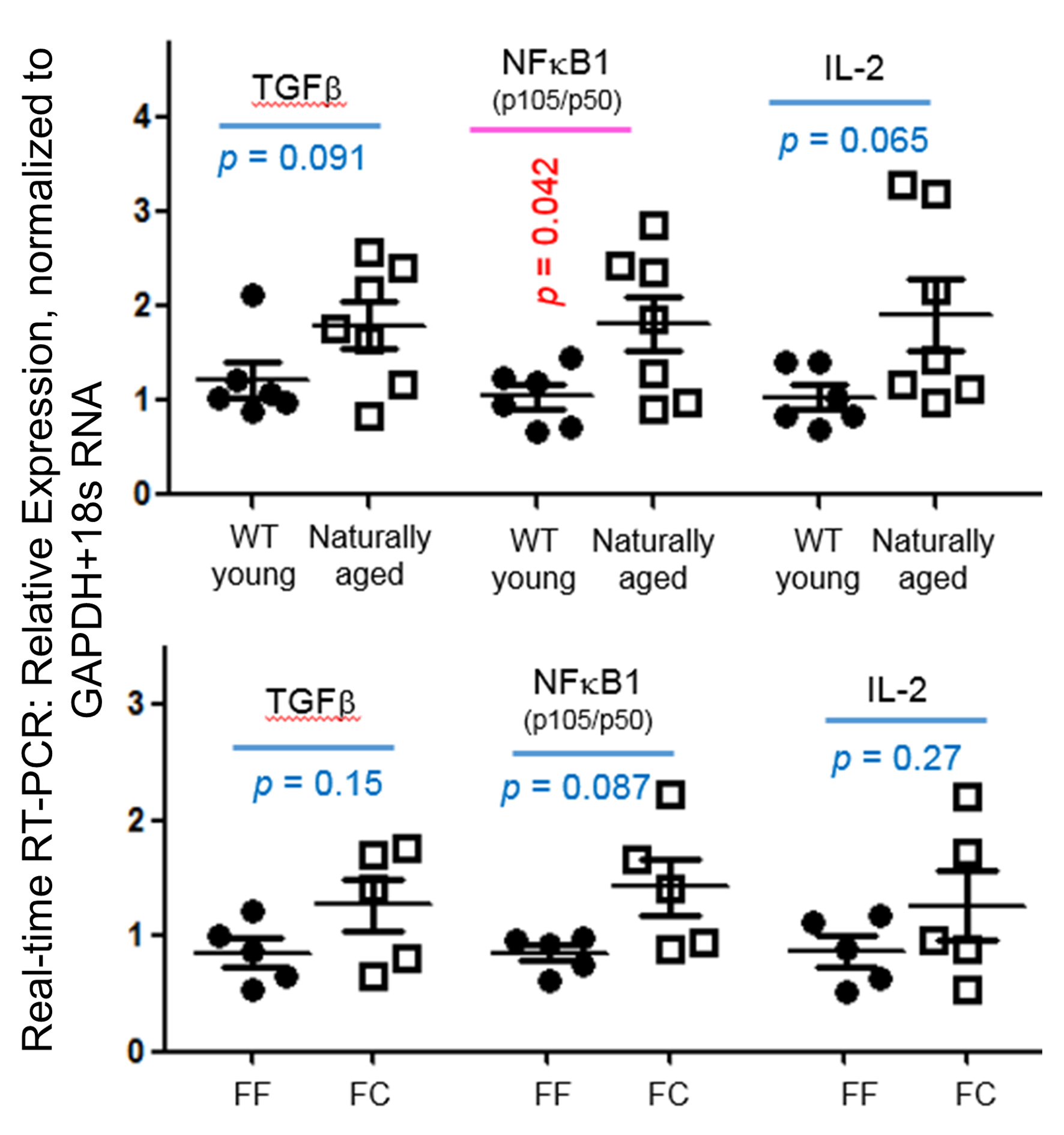

Supplement: S5 Fig — (Top) The mRNAs from the thymi of WT young and naturally aged mice; (Bottom) The mRNAs from thymi of FF (FoxN1fx/fx, without uCreERT) and FC (FoxN1fx/fx with CreERT, conditional knockout) mice (both groups treated with TM x3). A TaqMan-based real-time RT-PCR was conducted with TaqMan primers and probes to TGFβ, NF-κB (p105/p50), and IL-2, along with house-keeping genes, GAPDH and 18sRNA, for normalization. A Student t-test was used to determine statistical significance between groups. Data are expressed as mean ± SEM. Each symbol represents an animal. Underlying data used in the generation of this figure can be found in S1 Data. CreERT, ubiquitous promoter-driven Cre-recombinase and estrogen-receptor fusion protein; FC, FoxN1fx/fx/CreERT; FoxN1, Forkhead box protein N1; FoxN1fx/fx, loxp-flanked FoxN1 gene; IL-2, interleukin-2; NF-κB, Nuclear Factor kappa Beta; RT, reverse transcribed; PCR, polymerase chain reaction; tTreg, thymic regulatory T cell. (TIF) [file pbio.2003352.s005.tif]

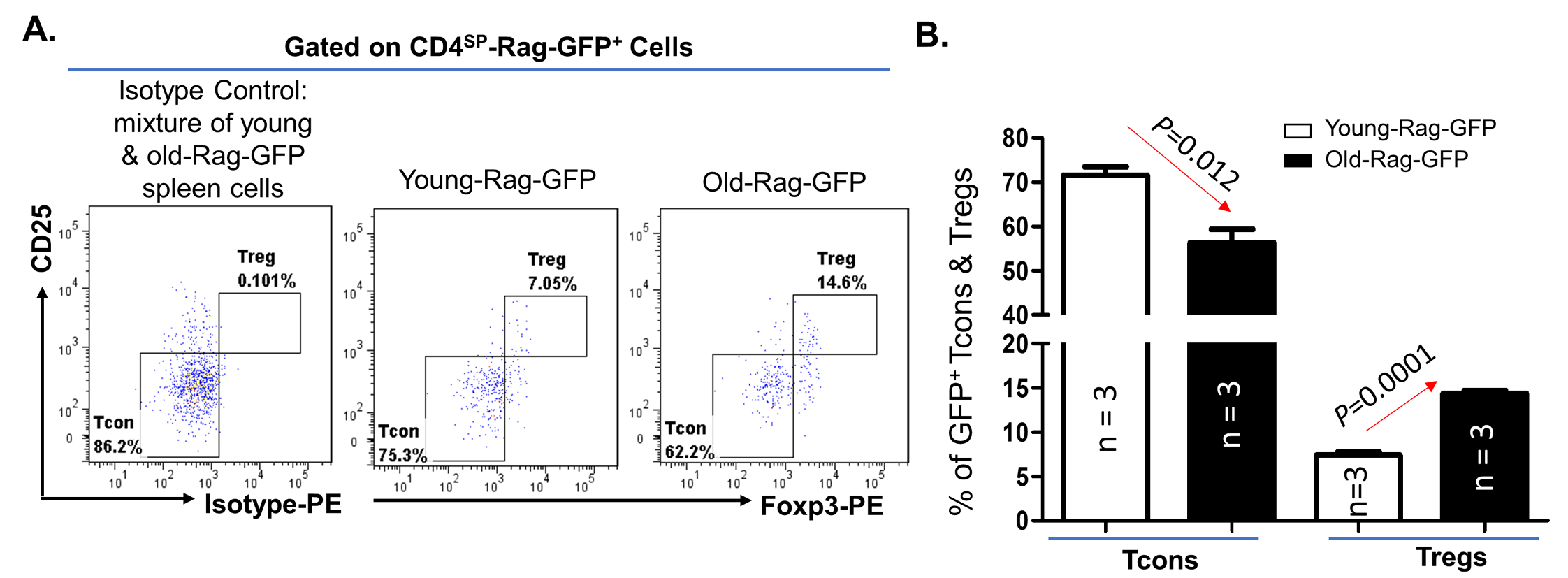

Supplement: S6 Fig — (A) Flow cytometric gate strategy shows gates of splenic Treg cells and Tcon cells from isotype control sample (mixture of young and old spleen cells stained with isotype control antibody for FoxP3); Young and old Rag-GFP reporter mice. (B) A summary of percentages of splenic Tcon cells and Treg cells in young and old mice, showing decreased RTE Tcon cells and increased RTE Treg cells in the old spleens. Underlying data used in the generation of this figure can be found in S1 Data. FoxP3, forkhead box P3; GFP, green fluorescent protein; RTE, recent thymic emigrant; Tcon, conventional T cell; Treg, regulatory T cell; tTcon, thymic conventional T cell; tTreg, thymic regulatory T cell. (TIF) [file pbio.2003352.s006.tif]
